# Endometrial cancer in women with abnormal uterine bleeding: Data mining classification methods 

**DOI:** 10.22088/cjim.14.3.526

**Published:** 2023

**Authors:** Farah Farzaneh, Azadeh Jafari Ashtiani, Mohammad Hashemi, Maryam Sadat Hosseini, Maliheh Arab, Tahereh Ashrafganjoei, Shaghayegh Hooshmand Chayjan

**Affiliations:** 1 Preventative Gynecology Research Center, Shahid Beheshti University of Medical Sciences, Tehran, Iran; 2 Faculty of Computer Science and Engineering, Shahid Beheshti University, Tehran, Iran

**Keywords:** Endometrial cancer, Artificial intelligence, Machine learning.

## Abstract

**Background:**

Over the last decade, artificial intelligence in medicine has been growing. Since endometrial cancer can be treated with early diagnosis, finding a non-invasive method for screening patients, especially high-risk ones, could have a particular value. Regarding the importance of this issue, we aimed to investigate the risk factors related to endometrial cancer and find a tool to predict it using machine learning.

**Methods:**

In this cross-sectional study, 972 patients with abnormal uterine bleeding from January 2016 to January 2021 were studied, and the essential characteristics of each patient, along with the findings of curettage pathology, were analyzed using statistical methods and machine learning algorithms, including artificial neural networks, classification and regression trees, support vector machine, and logistic regression.

**Results:**

Out of 972 patients with a mean age of 45.77 ± 10.70 years, 920 patients had benign pathology, and 52 patients had endometrial cancer. In terms of endometrial cancer prediction, the logistic regression model had the best performance (sensitivity of 100% and 98%, specificity of 98.83% and 98.7%, for trained and test data sets respectively,) followed by the classification and regression trees model.

**Conclusion:**

Based on the results, artificial intelligence-based algorithms can be applied as a non-invasive screening method for predicting endometrial cancer.

One of the most common cancers among women is endometrial cancer, which is the fourth most common cancer in the United States after breast, lung and colorectal cancers (1). Various causes can be considered risk factors for endometrial cancer, high levels of estrogen, high blood pressure, premature menstruation, late menopause, tamoxifen use, nulliparity, Lynch syndrome and old age are risk factors (55≤) (1-6). According to the American Cancer Society, all women over the age of 65 should be aware of the risk factors and symptoms of endometrial cancer so that they can be referred for further evaluation if any symptoms occur. Abnormal uterine bleeding is one of the most common manifestations of endometrial cancer, especially after menopause, which should be evaluated (1). Endometrial cancer is on the rise due to increased life expectancy and the prevalence of obesity (2). Evidence suggests that endometrial adenocarcinoma is more likely to be treated than other female cancers; observing the early signs of abnormal vaginal bleeding causes patients to see a specialist more quickly and receive treatment in the early stages of the disease (7, 8). Although abnormal uterine bleeding is the most common symptom, it is not a good indicator of endometrial cancer. Only 10% of women with cancer present with this symptom, and 90% of women undergoing aggressive diagnostic procedures do not have cancer (9). 

Numerous studies have been conducted to find endometrial cancer screening tools at the primary care level to determine which patients should be evaluated further (9). Artificial intelligence is a powerful mathematical tool and can be used significantly to promote public health (9). Machine learning is a branch of artificial intelligence through which a computer system learns potential patterns using existing data and helps identify complex patterns (10). It is a scientific order that concentrates on how computers learn from data (11).

Conventional biostatistical methods are not suitable for managing complex data (10, 12). The benefits of using machine learning over traditional statistical methods include flexibility and scalability (12). Machine learning algorithms are used to analyze various and complex data types in large quantities and are used to predict disease risk, classification, prognosis, diagnosis, and suitable treatment (12). In recent years, advanced classification techniques like artificial neural networks, classification and regression trees, Support Vector Machine, and logistic regression have been used widely for the prediction of many diseases, including cancers (13-21). Since endometrial cancer can impose an economic burden on the health system and it is very important to address this issue, in this study we have tried to use statistical approaches to investigate the importance of factors associated with endometrial cancer in women with abnormal uterine bleeding. Therefore, the present study aimed to utilize features and build predictive models to estimate a function for mapping input (features) to output (cancerous or non-cancerous). We can eventually indicate the best classification model as a non-invasive predictive and screening tool for endometrial cancer.

## Methods

**Study design: **In this cross-sectional study, 972 patients with abnormal uterine bleeding who were referred to the gynecology clinic of Imam Hossein Hospital in Tehran, Iran, were examined from 2016 to 2021. After receiving the ethical approval from the ethics committee of the Vice-Chancellery of Research at Shahid Beheshti University of Medical Sciences (code: IR.SBMU.RETECH.REC.1400.461) and informed written consent from patients, they were entered into the study. Inclusion criteria are women with abnormal uterine bleeding at reproductive or menopausal age who underwent physical examination, radiological and laboratory assessment, and endometrial sampling through dilatation and curettage. Pathological results were reported as benign for 920 and malignant for 52 patients, respectively. Then, based on the data and with the help of artificial intelligence algorithms such as Artificial Neural Networks and traditional machine learning models such as Logistic Regressions, Classification and Regression Trees, and Support Vector Machine, we evaluate the risk factors connected to endometrial cancer and compare the predictive accuracy of these algorithms with each other. Thus, some features were rerecorded for each case within the existing database, including age, body mass index, type of abnormal uterine bleeding, size of uterus in physical exam, history of other diseases, history of pregnancy, menarche age, menopausal age, menopausal status, family history of cancer, tamoxifen use, and endometrial thickness with the size of the uterus on ultrasound. Present study utilized these features and built predictive models to estimate a function for mapping input (features) to output (cancerous or non-cancerous).

**Statistical analysis: **Quantitative data were presented as mean and standard deviation or median and interquartile range, and qualitative data were presented as frequency and percentage. The chi-square test, independent sample t-test, and Mann-Whitney test were applied for univariate analysis; all the variables with p<0.05 and frequency more significant than 10% were included further in classification models. All statistical analyses were performed using a 0.05 significance level.

In the present study, four different methods were selected to determine and compare their predictive accuracy in the diagnosis of endometrial cancer. The existing datasets were divided into two parts: training data and test data (with a ratio of 4 to 1). In the training phase, the model tries to find the best parameters and weights for the function. The goal is to have the best classification with the least error rate on the test set. The following methods were implemented in Python programming language and Scikit-learn framework as an efficient tool for predictive data analysis.

**Logistic Regression: **A logistic regression model predicts dependent data variables by analyzing the relationship between one or more existing independent features. It is widely used to predict several diseases; hence it is of great importance to be implemented. In addition, the final logistic regression model was assessed by Hosmer and Lemeshow test.

**Classification and Regression Trees: **Classification and regression trees were used to classify data into two categories (22). Moreover, they can identify characteristics and develop rules; hence, they are known as an explanatory method valuable for experts in medicine. The representation for the classification and regression trees model is a binary tree, and each root node represents a single input variable (x) and a split point on that variable. The tree's leaf nodes contain an output variable (y) which is used to make a prediction.

**Figure 1a:**
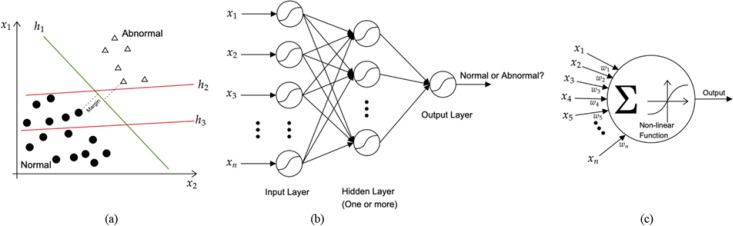
Support Vector Machine model. Hyper-plane h3 cannot separate abnormal cases from normal ones. Hyper-plane h2 does, but only with a small margin. On the other hand, the Support Vector Machine result which is the hyper-plane h1 separates them with the maximal margin. b: A typical structure of Multi-Layer Perceptron, a common variant of Artificial Neural Networks for a binary classification problem (Distinguishing endometrial cancer from normal cases). c: A diagram of a single artificial neuron. (x_1 - x_n) are inputs multiplied by weights (w_1 - w_n), then they are aggregated with the summation, and the outcome is passed through an activation function (non-linear function e.g., sigmoid) to produce the neuron output

**Support Vector Machine: **Support Vector Machine is a supervised learning approach that organizes data into categories and is a machine learning algorithm that analyzes data for classification. By distinguishing hyper-planes in a high-dimensional feature space, the goal of a Support vector machine is to construct a computationally efficient way of learning (23). Numerous hyper-planes could be used to classify two sets of data. The hyper-plane with the most significant margin should be picked as the best option. The margin is the maximum width that the boundary can increase before colliding with a data point. The data points that the margin pulls up are referred as support vectors (figure 1-a). As a result, the support vector machine's aim was to determine the best hyper-plane for separating categories of target vectors on opposite sides of the plane (23). 

**Artificial Neural Network: **Artificial neural networks are computational networks inspired by biology. It is a computer modeling approach that learns from examples through iterations without requiring prior knowledge of the relationships between process parameters. As a result, it can deal with uncertainty, noisy data, and non-linear correlations, unlike many traditional methods based on linear techniques. Artificial neural networks are well suited for classification and prediction tasks in practical circumstances because of their capacity to learn from a specific data set (24-26).

We concentrated on multilayer perceptron (24, 25) with back propagation learning algorithms among the numerous algorithms used in artificial neural networks in this study. The multilayer perceptron is a supervised artificial neural network that has three types of layers: input, hidden, and output. They are the most often used artificial neural network for a wide range of issues. The multilayer perceptron is made up of a network of artificial neurons that are coupled so that the output of one neuron becomes the input of one or more other neurons. Specifically, an input layer of neurons receives the input data, one or more hidden layers, and eventually an output layer that provides the network's output. A typical architecture of a multi-perceptron model has been drawn (figure 1-b). The weighted input values to a single neuron are aggregated using a vector to scalar function like summation (i.e., $y_{j}=\sum_{i}w_{\mathrm{ij}}x_{i}$) averaging, input maximum or mode value produce a single input value to the neuron. The neuron then utilizes an activation function to produce its output after calculating the input value (and consequently the input signals for the next layer). The activation function transforms the input value of the neuron. A sigmoid, hyperbolic-tangent or other nonlinear function is commonly used in this transformation. The structure of a single neuron is depicted in (figure 1-c).

## Results

Nine hundred seventy-two patients with a mean age of 45.77 ± 10.70 years (ranging from 25 – 85 years) were recruited in this study, of which 52 (5.3%) cases were diagnosed with endometrial cancer. A significant difference was observed between the two groups (benign and malignant) with regard to age (p<0.001), body mass index (p<0.001), menarche age (p<0.001), history of pregnancy (P=0.032), uterus size in bimanual exam (P=0.032), post-menopausal bleeding (p<0.001), menometrorrhagia (P=0.016), menorrhagia (P=0.002), metrorrhagia (P=0.003), history of cancer in patients (p<0.001), diabetes mellitus (p<0.001), hypertension (p<0.001), polycystic ovarian disease (P=0.001), hypothyroidism (P=0.009), menopause (p<0.001), menopause age (P=0.008), and endometrial thickness (p<0.001).Statistically significant differences were not observed between the two groups regarding family history of cancer, Tamoxifen use, and infertility (table 1). 

**Table 1:** Study subjects' characteristics according to the endometrial cancer

**Variables**	**Benign, N**	**Malignant, N**	**P- Value**
**Age (year), mean±standard deviation**	45.01 ± 10.37	53.40 ± 13.22	<0.001
**Body Mass Index (kg/m** ^2^ **), Mean±Standard deviation**	28.75 ± 4.78	31.95 ± 3.43	<0.001
**Menarche age(year), Mean±Standard deviation**	13.32 ± 1.22	10.60 ± 0.569	<0.001
**History of pregnancy (%)**	829 (90.1)	42 (80.8)	0.032
**Large uterus size in bimanual exam (%)**	363 (39.5)	32 (61.5)	0.002
**Family history of cancer (%)**	38 (4.1)	0 (0)	0.259
**First degree (%)**	26 (2.8)	0 (0)	
**Second degree (%)**	12 (1.3)	0 (0)	
**Tamoxifen use (%)**	17 (1.8)	2 (3.8)	0.270
**Menometrorrhagia**	144 (15.7)	2 (3.8)	0.016
**Menorrhagia**	214 (23.3)	3 (5.8)	0.002
**Metrorrhagia**	179 (19.5)	2 (3.8)	0.003
**Post-Menopausal Bleeding (%)**	209 (22.7)	43 (82.7)	<0.001
**Spotting**	174 (18.9)	2 (3.8)	0.003
**History of any kind of cancer in patients (%)**	99 (10.8)	16 (30.8)	<0.001
**Diabetes Miletus (%)**	77 (8.4)	16 (30.8)	<0.001
**Hypertension (%)**	128 (13.9)	33 (63.5)	<0.001
**Hypothyroidism (%)**	90 (9.8)	11 (21.2)	0.009
**Infertility (%)**	45 (4.9)	4 (7.7)	0.327
**Polycystic Ovarian Disease (%)**	6 (0.7)	4 (7.7)	0.001
**Menopause (%)**	342 (37.2)	45 (86.5)	<0.001
**Menopause age** ** (year), mean±Standard deviation**	50.74 ± 4.67	52.76 ± 5.79	0.008
**Uterus size in sonography (mm), median (Interquartile Range)**	78 (65 - 87)	80 (71.25 - 89)	0.096
**Endometrial Thickness in sonography (mm),** **median (Interquartile Range)**	10 (8 - 13)	56.5 (19.5 – 31.75)	<0.001

**Figure 2:**
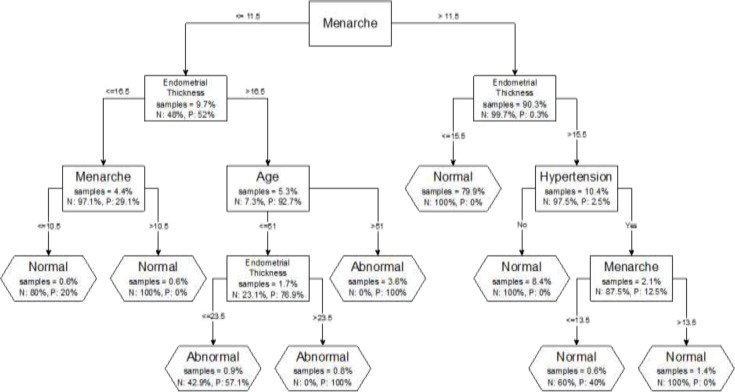
The classification and regression tree model for the prediction of endometrial cancer

According to multiple logistic regression result, the independent risk factors were higher body mass index (odds ratio=1.34; 95%confidence interval: 1.07 – 1.68), lower menarche age (odds ratio=0.157; 95%confidence interval: 0.068 – 0.363), larger endometrial thickness (odds ratio=1.31; 95%confidence interval: 1.16 - 1.49) and being hypertensive (odds ratio=15.25; 95%confidence interval: 1.02 – 227.01), data are shown in table 2. Nine rules were extracted according to the classification and regression trees result (figure 2). The most important variable was menarche age, followed by endometrial thickness and patients’ age, for those with menarche age younger than 11.5 years (category 1) and endometrial thickness and hypertension for those whose menarche age was older than 11.5 years (category 2). Subjects presented in category 1 had a higher risk of endometrial cancer if they had an endometrial thickness size of greater than 16.5 mm and they were older than 51.1 years (P=100%) also, younger patients who had endometrial thickness size of (23.5 – 51.1) mm were more prone to endometrial cancer risk (P=100%), while the risk of endometrial cancer was lower among younger patients whose endometrial thickness size was in the range of 16.5 – 23.5 mm (P=57.1%). Other rules were in favor of normal endometrium. The best performance of all methods was attributed to the logistic regression model (sensitivity of 100% and 98%, specificity of 98.83% and 98.7%, positive predictive value of 82.97% and 82.85%, and the negative predictive value of 100% and 99.89% for trained and test data set respectively), followed by the classification and regression trees model (table 3).

**Table 2:** Multiple logistic regression results of factors affecting endometrial cancer

**Variable**	**Odds Ratio**	**95% Confidence Interval**	**P-Value**
**Age**	1.09	0.933 - 1.27	0.282
**Body Mass Index**	1.34	1.07 - 1.68	0.012
**Menarche age**	0.157	0.068 - 0.363	<0.001
**Menopause**	0.990	0.003 - 24.99	0.997
**Pregnancy**	0.248	0.004 - 15.11	0.506
**Post-Menopausal Bleeding**	7.95	0.068 - 931.7	0.394
**Large uterus size**	2.63	0.270 - 25.69	0.404
**Endometrial thickness**	1.31	1.16 - 1.49	<0.001
**Diabetes Miletus**	1.68	0.044 - 64.72	0.780
**Hypertension**	15.25	1.02 - 227.01	0.048
**Infertility**	2.63	0.026 - 266.7	0.682
**Polycystic Ovarian Disease**	1.28	0.006 - 283.2	0.934
**History of cancer**	2.22	0.001 - 2888	0.912

**Table 3:** Performance indices of the four applied classification models (train/test)

**Performance indices**	**Support vector Machine**	**Logistic Regression**	**Classification and egression trees**	**Artificial Neural Network**
**Sensitivity**	89.45/86.55	100.0/98.0	92.31/88.18	89.92/88.55
**Specificity**	99.59/99.46	98.83/98.7	99.57/98.91	99.86/99.78
**Positive predictive value**	92.59/91.33	82.97/82.85	92.57/83.51	97.41/96.67
**Negative predictive value**	99.4/99.24	100.0/99.89	99.57/99.35	99.43/99.35
**False positive rate**	0.41/0.54	1.17/1.3	0.43/1.09	0.14/0.22
**False negative rate**	10.55/13.45	0/2.0	7.69/11.82	10.08/11.45
**Overall accuracy**	99.05/98.77	98.89/98.66	99.18/98.35	99.33/99.18

P: (Positive) malignant cases; N: (Negative) benign cases

## Discussion

**Summary of Main Results**: Endometrial cancer is one of the pivotal issues in gynecology oncology, so a predictive model can help clinicians assess patients properly. This study specified which factors are more significant in developing endometrial cancer and described four models for predicting endometrial cancer by employing machine learning algorithms. In our study, logistic regression had more sensitivity than classification and regression trees, support vector machine, and artificial neural networks. However, there were no significant differences in overall accuracy among them for endometrial cancer risk calculation, and considering all epidemiological criteria, logistic regression and classification and regression trees had better performance.

**Results in the Context of Published Literature: **Using machine learning algorithms in medicine is expanding and applied to evaluate various diseases. The study results by Khoury et al. in 2019 showed that machine learning methods could be used to diagnose patients with Parkinson's disease and differentiate this disease from other neurodegenerative diseases such as Huntington's and Amyotrophic Lateral Sclerosis (19). A meta-analysis by Nindrea et al. (2018) found that the support vector machine algorithm's accuracy value in calculating breast cancer risk was more than other machine learning algorithms (20). In recent years machine learning has provided valuable information in the field of gynecological cancers. In 2015 Enshaei et al. found the artificial neural networks as a prognostic and predictive model for ovarian cancer (27).

A 2019 study by Kawakami et al. demonstrated that artificial intelligence-based algorithms could help physicians to select the proper treatment for ovarian cancer patients. (10). 

Pergialiotis et al. (2018) described that artificial neural network had more sensitivity and specificity than classification and regression trees and logistic regression for the prediction of endometrial cancer in postmenopausal women, respectively (9). Hutt et al. (2021) reported that neural network models were useful tools in the prediction and risk calculation of endometrial cancer (28). Artificial intelligence can use radiological images to predict some evidence about cancer. A 2020 study by Dong et al. determined that artificial intelligence could help the radiologist interpret magnetic resonance images or be an acceptable alternative for assessing the depth of preoperative myometrial invasion in patients with stage one endometrial cancer (29). Contrary to the advantages, some restrictions of using artificial intelligence include clinicians' perception of machine learning models, ethical challenges, and data collecting (12).

**Strengths and Weaknesses: **This study determined crucial risk factors for endometrial cancer using statistical approaches and then made models for predicting endometrial cancer using machine learning algorithms. On the other hand, these models can be used to decrease the probability of endometrial cancer by selecting at-risk patients and applying preventive strategies. We had limitations to access electronic records of other centers to increase our data set. Moreover, we could hardly find medical students or doctors who know artificial intelligence. 

**Implications for Practice and Future Research: **Through multi-central studies, we could increase our data sets to reach better results and improve models' sensitivity. The artificial neural networks, support vector machine, classification and regression trees, and logistic regression models recruited in this study had acceptable and close overall accuracy and may help diagnose endometrial cancer with less invasive and expensive methods. It seems that, in the computer and digital era, physicians need to do more research based on artificial intelligence and its contributing role in medical science and diagnosing and treating diseases.
